# Data on differentially expressed proteins in retinal emmetropization process in guinea pig using integrated SWATH-based and targeted-based proteomics

**DOI:** 10.1016/j.dib.2018.08.119

**Published:** 2018-08-31

**Authors:** Sze Wan Shan, Dennis Yan-yin Tse, Bing Zuo, Chi ho To, Quan Liu, Sally A. McFadden, Rachel Ka-Man Chun, Jingfang Bian, King Kit Li, Thomas Chuen Lam

**Affiliations:** a Laboratory of Experimental Optometry, Centre for Myopia Research, School of Optometry, the Hong Kong Polytechnic University, Kowloon, Hong Kong; b State Key Laboratory of Ophthalmology, Zhongshan Ophthalmic Center, Sun Yat-Sen University, Guangzhou, China; c School of Psychology, Faculty of Science, University of Newcastle, Callaghan, New South Wales, Australia

## Abstract

Myopia is generally regarded as a failure of normal emmetropization process, however, its underlying molecular mechanisms are unclear. Retinal protein profile changes using integrated SWATH and MRM-HR MS were studied in guinea pigs at 3- and 21-days of age, where the axial elongation was significantly detected. Differential proteins expressions were identified, and related to pathways which are important in postnatal development in retina, proliferation, breakdown of glycogen-energy and visual phototransduction. These results are significant as key retinal protein players and pathways that underlying emmetropization can be discovered. All raw data generated from IDA and SWATH acquisitions were accepted and published in the Peptide Atlas public repository (http://www.peptideatlas.org/) for general release (Data ID PASS00746). A more comprehensive analysis of this data can be obtained in the article “Integrated SWATH-based and targeted-based proteomics provide insights into the retinal emmetropization process in guinea pig” in Journal of Proteomics (Shan et al., 2018) [1].

**Specifications Table**

**Table:** 

Subject area	Biology
More specific subject area	Biology in eye development and emmetropization
Type of data	Table, graph, figure
How data was acquired	SWATH Mass Spectrometry*;* MRM-HR; Quadrupole Time-of-Flight TripleTOF® 6600 mass spectrometer (SCIEX); searched against the UniProt database (organism ID: 10141)
Data format	analyzed
Experimental factors	Age
Experimental features	Retinal proteins were isolated from guinea pig at 3 and 21 days old (*n* = 10). The protein lysate was used for differentially expressed proteins identification using SWATH mass spectrometry and high resolution Multiple Reaction Monitoring (MRM-HR) MS for validation.
Data source location	Centre for Myopia Research, School of Optometry, the Hong Kong Polytechnic University, Kowloon, Hong Kong
Data accessibility	All raw data generated from IDA and SWATH acquisitions were accepted and published in the Peptide Atlas public repository (http://www.peptideatlas.org/) for general release (Data ID PASS00746).

**Value of the data**

• These data combined and described the use of the integrated SWATH MS and MRM-HR mass spectrometry to assess the biological mechanism(s) during emmetropization in guinea pigs.
• Novel pathways were identified first reported during emmetropic eye growth in guinea pig.
• Differential protein changes were identified in the retina between 3- and 21-days of age in guinea pigs which uncover the most significant molecular events in normal eye growth.

## 1 Data

**Fig. 1:**
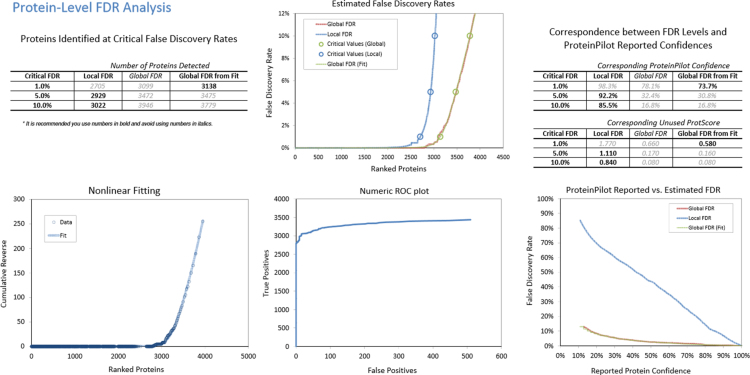
FDR analysis of combined IDA library of retina at protein level generated by Protein Pilot software.

**Fig. 2:**
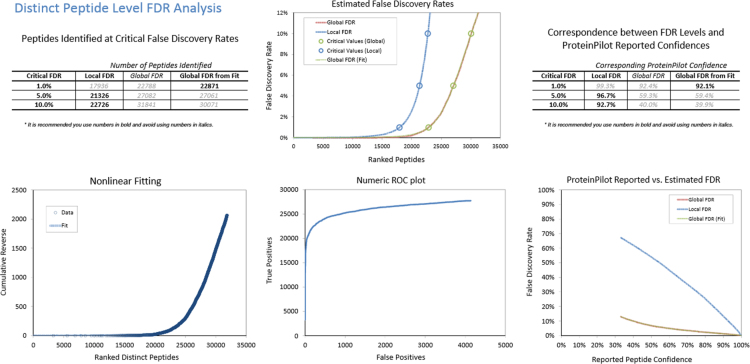
FDR analysis of combined IDA library of retina at peptide level generated by Protein Pilot software.

Protein identification was performed by using ProteinPilot 5.0 (PP5.0, Sciex Framingham, MA) software and Fig. 1, Fig. 2 show the protein and peptide FDR analyses result, respectively. In the Table S1, it shows the full list of a total of 3138 (at 1% FDR) non-redundant protein IDs that identified in the combined ion library. All shared proteins (Table S2) with fold changes, *p*-value and confidence values which found by SWATH MS were uploaded to the iPATHWAYGuide (http://www.advaitabio.com/) for evaluation. 48 isolated proteins were found to be differentially expressed after cutting off the peptide matching (≤ 1 peptide), confidence levels (≥ 75%), and fold changes (≥ 0.43 in Log 2 fold change).

## 2 Experimental design, materials and methods

### 2.1 Animals

Animal housing and biometric measurements performed were similar to our previous work [2]. Ten pigmented guinea pigs (Carvia porellus, Dunkin Harley) were raised in their natural litter with their mother. Each litter was housed in a box (65 × 45 × 20 cm) in a temperature-controlled room, and water and food were freely available. Lighting was provided by three white light-emitting diodes (1400 mA, Luxeon III Star; Philips Lumileds Lighting Company, San Jose, CA) evenly diffused through a Perspex barrier located 20 cm above each box. The luminance in the center of each holding box was 500 lx on a 12/12 h light/dark cycle. All procedures were approved by the University of Newcastle in accordance with Australian legislative requirements and were in accordance with NIH Guidelines. Retina were collected from five animals at postnatal day 3 (PN3) and five at postnatal day 21 (PN21) with similar weights within a group (*n* = 10 eyes for each time point).

### 2.2 Retinal protein extraction

The retinal protein extraction protocol was performed similar to our earlier publications [3], [4]. After deep anesthesia (injection of 130 mg/kg pentobarbitone sodium into the heart in animals anesthetized with 1.5% isoflurane in oxygen) the eyes were rapidly enucleated within 5 minutes. After removing the extraocular tissues, a circular cut was made around the limbus and the anterior segment, crystalline lens, and vitreous were discarded. The retina was dissected free of retinal pigment epithelium, and carefully peeled off from the posterior hemisphere, immediately frozen in liquid nitrogen, and stored at − 80 °C before further analysis.

Each frozen retinal sample was homogenized in a liquid nitrogen cooled Teflon chamber (Mikrodismembrator, B. Braun Biotech International, Germany) with 200 µl customized lysis buffer (containing 7 M urea, 2 M thiourea, 30 mM tris, 2% CHAPS, 1% ASB14) for 7 minutes at 1600/rpm. The homogenized retina sample was stored at 4 °C for 30 min. Then, after centrifuging at 16.1 × 1000*g* for 30 min at 4 °C, the supernatant was collected. The total protein concentrations were determined with the 2-D Quant Kit (Amersham Biosciences, US).

### 2.3 Pooling of retinal samples and enzymatic digestion for LC–MS/MS

Five individual retinal samples from each group (3 days Right eye, PN3_R; 3 days Left eye, PN3_L; and 21 days Right eye, PN21_R and 21 days Left eye, PN21_L) were pooled to form representative lysates. The resulting total 40 µg mixed retinal protein was reduced with 8 mM Dithiothreitol (DTT) (45 min at 37 °C), alkylated with 20 mM iodoacetamide (30 min in dark at 25 °C), and 4× volume of cold acetone was added to the solution with brief vortexing. The mixture was incubated at − 20 °C overnight, then centrifuged at 16,000*g* for 20 min at 4 °C. The pellet was then washed with 100 µL of 80% acetone with brief vortexing and centrifuged at 16,000*g* for 10 min at 4 °C. The protein pellet was dissolved in 8 M urea and 25 mM ammonium bicarbonate and then diluted to 4 M urea with 25 mM ammonium bicarbonate. A Bradford Protein Assay (Bio-Rad) was used to determine protein concentration. Thirty micrograms protein of each sample were diluted to 1 M urea with 25 mM ammonium bicarbonate. Trypsin was added at 1:25 (enzyme:protein) ratio w/w, and the mixture was incubated overnight at 37 °C. Trypsin-digested peptides were acidified with 0.5% formic acid prior to LC–MS/MS.

### 2.4 Setting of LC–MS/MS

A hybrid Quadrupole Time-of-Flight TripleTOF® 6600 mass spectrometer (SCIEX) was used for both IDA (information-dependent acquisition) and SWATH-MS analyses. The trap column (200 μm × 0.5 mm) and the analytical column (75 μm × 15 cm) were packed with 3 μm ChromXP C18 medium. The samples were loaded at a flow rate of 3 μl min^−1^ for 15 min with loading buffer, 2% (v/v) acetonitrile with 0.1% (v/v) formic acid, and eluted from the analytical column at a flow rate of 300 nl min^−1^ in a mixture of solvent A and B with a linear gradient of 5% solvent B to 35% solvent B in 120 min. Solvent A was composed of 2% (v/v) acetonitrile with 0.1% (v/v) formic acid. Solvent B was composed of 98% (v/v) acetonitrile with 0.1% (v/v) formic acid. The column was regenerated by washing at 80% solvent B for 10 min and re-equilibrated at 5% solvent B for 17 min. Peptides were injected into the mass spectrometer using 10 µm SilicaTip electrospray emitters (New Objective Cat. No. FS360-20-10-N-20-C12), and the ion source was operated with the following parameters: ISVF = 2300; GS1 = 15; CUR = 30; and IHT = 120. The data acquisition mode in the information-dependent acquisition (IDA) experiments was set to obtain a high resolution TOF-MS scan over a mass range 350–1500 *m*/*z*, followed by 100 to 1800 *m*/*z* for MS/MS scans of 40 ion candidates per cycle, operating the instrument in high sensitivity mode. The selection criteria for the parent ions included the intensity, where ions had to be greater than 125 cps, with a charge state between 2 and 4. The dynamic exclusion duration was set for 18 s. Collision-induced dissociation was triggered by rolling collision energy. The ion accumulation time was set to 250 ms (MS) and to 80 ms (MS/MS). For SWATH MS-based acquisitions, the instrument was tuned to specifically allow a quadrupole resolution of 25 Da/mass selection. An isolation width of 25 Da was set in a looped mode over the full mass range (350–1500 *m*/*z*) scan, and 46 overlapping windows were constructed. An accumulation time of 80 ms was set for each fragment ion resulting in a total duty cycle of 3.78 s. The total LCMS running time for each IDA and DIA injection is 120 min.

For high-resolution MRM experiments, a 120-min effective gradient separation was used. Peptides (1 µg) were first loaded on a trap column (200 μm × 0.5 mm, C18) by loading buffer at 3 μl/min for 15 min, then separated on a nano-LC column (75 µm × 15 cm, ChromXP C18, 3 µm, 120 A) using an Ekisgent 415 nano-LC system. The LC separation was performed at under 300 nl/min using mobile phase A (0.1% formic acid and 5% acetonitrile in water) and B (0.1% formic acid in acetonitrile) with the following gradient: 0–1 min, 5% B; 1–91 min, 5–35%B; 91–95 min, 35%B; 95–101 min, 35–80%B; 101–111 min, 80%B; 111–113 min, 80%-5%B; and 113–130 min, 5%B.

### 2.5 Ion library generation for SWATH analysis

Two micrograms tryptic peptide from each of four pooled samples were used for the IDA experiments. The combined data from the IDA experiments of the four pooled samples were used to generate an ion library (.group file) for SWATH analysis. It was searched against the guinea pig Uniprot database [5] in ProteinPilot 5.0 (Sciex) software utilizing the Paragon algorithms with the following parameters: identification Sample Type, iodoacetamide Cys Alkylation, trypsin digestion, thorough search effort and with FDR analysis. The resulting protein pilot group file was used as the ion library file for all SWATH file processing and quantification.

### 2.6 SWATH acquisitions and processing

A 2 µg aliquot of tryptic peptide was used for each injection. Three technical replicates were performed for each of the four experimental groups. All the files from SWATH experiments were processed with PeakView 2.1. The ion library for SWATH analysis was selected from the IDA experiment. The maximum number of 3000 proteins were imported with unlabeled sample type. The retention time (RT) of all twelve runs was aligned by using 10 manually selected peptides with high intensity from 20 to 100 min of the run. The processing setting parameters were: 6 peptides per protein, 6 transitions per peptide, 95% peptide confidence threshold, 1% false discovery rate threshold, and a 5-min XIC extraction window with 75 ppm XIC width. After processing, the generated ion library and all individual SWATH files were uploaded to the OneOmics data environment hosted on the Basespace cloud (http://basespace.illumina.com) via CloudConnect micro application for PeakView.

### 2.7 MRM-HR data analysis

The targeted peptide transition lists and MRM-HR acquisition method were created by Skyline Software (MacCoss Lab, University of Washington, WA, USA) with manual screening. A TOF-MS scan in the mass range of 350–1250 Da with 0.25 s accumulation time was acquired followed by the product ion of targeted peptide precursors mass with 10 ms accumulation time. The integrated area of the ion pair for each peptide was achieved by MultiQuant 3.0 Software (SCIEX). The fragments to be used for each peptide were chosen from the top three high intensity fragments that had no apparent interference, and b and y ions were used. The peak areas for peptides were calculated by summing of the peak areas of their transition ions, and proteins were quantified by the total peak area of their corresponding peptides. The fold change was determined by comparing the mean value of the peak areas of peptides among the samples.

### 2.8 Bioinformatics analysis

Identified proteins (IDs) were converted into gene names using the batch id conversion tool on the Uniprot protein online database (http://www.uniprot.org/) for subsequent bioinformatics analysis. For the data filtering, parameters were set as follows: confidence-criterion was > 75% by the default setting in the OneOmics platform, fold change (≥ 1.4), *p*-values (≤ 0.05), and proteins required at least 2 matching peptides. The gene ontologies (biological processes and molecular functions) of all IDs was searched against the Gene Ontology database using PANTHER - Gene List Analysis (www.pantherdb.org) [6]. In addition, pathway analyses for protein-protein interactions of differentially expressed candidates were performed using the STRING v10 (Search Tool for the Retrieval of Interacting Genes/Proteins) online database [7] and a novel iPathwayGuide v1.1 (http://www.advaitabio.com) cloud based analysis. The GO for these differentially expressed proteins were further analyzed by the OneOmics platform.
