# Supplementary material for: Data on differentially expressed proteins in retinal emmetropization process in guinea pig using integrated SWATH-based and targeted-based proteomics
Source: Data Brief. 2018 Aug 31;21:1750–5. doi: 10.1016/j.dib.2018.08.119 (PMC6249517; doi:10.1016/j.dib.2018.08.119)
Supplement: Supplementary file 1 — Supplementary material [file mmc1.docx]

All the authors have no financial or commercial conflict of interest.
